# Feather holes and flight performance in the barn swallow *Hirundo rustica*

**DOI:** 10.1080/19768354.2018.1452294

**Published:** 2018-03-25

**Authors:** Piotr Matyjasiak, Paweł Boniecki, Maciej Fuszara, Mateusz Okołowski, Izabela Olejniczak

**Affiliations:** a Faculty of Biology and Environmental Sciences, Cardinal Stefan Wyszyński University in Warsaw, Warsaw, Poland; b Faculty of Christian Philisophy, Cardinal Stefan Wyszyński University in Warsaw, Warsaw, Poland; c Museum and Institute of Zoology, Polish Academy of Sciences, Warsaw, Poland

**Keywords:** Barn swallow, feather hole, feather quality, feather wear, flight performance

## Abstract

Feather holes are small (0.5–1 mm in diameter) deformities that appear on the vanes of flight feathers. Such deformities were found in many bird species, including galliforms and passerines. Holey flight feathers may be more permeable to air, which could have a negative effect on their ability to generate aerodynamic forces. However, to date the effects of feather holes on flight performance in birds remained unclear. In this study we investigated the relationship between the number of feather holes occurring in the wing or tail feathers and short term flight performance traits – aerial manoeuvrability, maximum velocity and maximum acceleration – in barns swallows, which are long distance migrating aerial foragers. We measured short-term flight performance of barn swallows in a standardized manner in flight tunnels. We found that acceleration and velocity were significantly negatively associated with the number of holes in the wing flight feathers, but not with those in the tail feathers. In the case of acceleration the negative relationship was sex specific – while acceleration significantly decreased with the number of feather holes in females, there was no such significant association in males. Manoeuvrability was not significantly associated with the number of feather holes. These results are consistent with the hypothesis that feather holes are costly in terms of impaired flight. We discuss alternative scenarios that could explain the observed relationships. We also suggest directions for future studies that could investigate the exact mechanism behind the negative association between the number of feather holes and flight characteristics.

## Introduction

**Figure 1.**
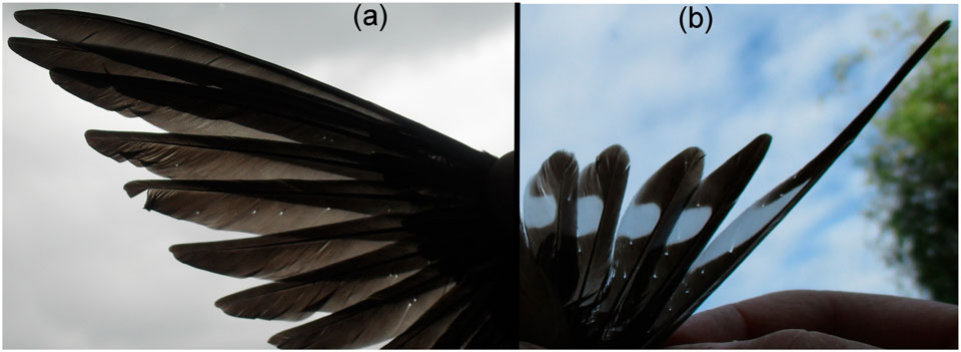
Feather holes in flight feathers of the wing (a) and tail (b) of the barn swallow.

Feathers are integument structures that have evolved in theropod dinosaurs and nowadays are representative of birds. The quality of bird feathers is important for such functions of the plumage as flight performance, mechanical protection, thermal insulation, water repellence, and visual communication (Jenni and Winkler 1994). Full-grown feathers are dead structures, and unlike other keratinized structures, like claws or hair, they are not subject to regeneration. Hence old feathers must be totally replaced during moult. Feathers are gradually damaged between two moults, which is particularly true for the flight feathers. Among the most common examples of feather structural damage there are mechanical abrasion (feather wear or breakage), due to rubbing against objects in the environment or air particles, and feather holes (Burtt 1986; Jenni and Winkler 1994). Feather holes are small (0.5–1 mm in diameter) deformities that appear on the vanes of flight feathers during the period between two moults (Figure 1). Within the feather holes barbules are absent and barbs can be cut (see e.g. Vas et al. 2008, p. 1439; Stenkewitz et al. 2017, p. 2). These feather deformities are found in many different bird species, including rock ptarmigan *Lagopus muta* (Stenkewitz et al. 2017), barn swallow *Hirundo rustica* (Møller 1991), and other passerines (Vas et al. 2008; Vágási 2014). So far it was thought that feather holes are the traces of the grazing activity of chewing lice (Phthiraptera: Amblycera, Ischnocera; Møller 1991; Vas et al. 2008; Møller et al. 2004a, 2004b; Stenkewitz et al. 2017). However, recently it has been suggested that chewing lice may not be the main causative agent of feather holes and other factors, such as keratinolytic microorganisms and developmental defects in the structure of feathers, can be involved (Vágási 2014).

There is a sound body of evidence for the locomotion costs of feather abrasion or feather replacement (Swaddle et al. 1996; Chai 1997; Swaddle and Witter 1997; Chai and Dudley 1999; Lind 2001; Lind and Jakobsson 2001; Williams and Swaddle 2003; Tomotani et al. 2017). The studies reported that birds undergoing moult of their flight feathers exhibited a reduced forward flight velocity, aerial manoeuvrability and/or angle of escape flight trajectory. Birds were able to compensate to a greater or lesser extent the aerodynamic disadvantage of moult gaps present in their wings by increasing flight muscles and/or by reducing body weight (e.g. Williams ans Swaddle 2003). It was also found that birds that acquired fresh flight feathers performed significantly better during flight tests compared to the same individuals before moult (e.g. Chai and Dudley 1999; Williams and Swaddle 2003). This discovery points to the advantage of high quality plumage in relation to locomotion (see also Echeverry-Galvis and Hau 2013). The number of holes present in flight feathers is considered one of indicators of the functional quality of these feathers (Pap et al. 2007; Vágási 2014). Holey flight feathers can be less airtight thus impairing the ability of wings to maintain pressure difference between the air above and below the wing, which is necessary for the generation of aerodynamic forces through wings (Videler 2005). This can be detrimental to flight performance. Barbosa et al. (2002) have investigated the effect of feather holes on flight in the barn swallow and concluded that feather holes do not produce mechanical constrains on flight. According to other studies that also were carried out on the barn swallow, the feather hole count was significantly negatively correlated with migratory performance and annual survival rate (Møller et al. 2004a; Pap et al. 2005). These findings suggest that holey flight feathers can affect flight in this highly aerial and long distance migrating bird species. We are unaware of any subsequent studies testing for the effects of flight feather holes on flight performance.

In the present study we aimed at investigating the relationship between the number of holes present in flight feathers of the wings and tail and the short-term flight performance (aerial manoeuvrability, maximum acceleration and maximum velocity) in the barn swallow. Barn swallows are small (ca. 20 g), aerially insectivorous long-distance migratory birds that spend winter in the southern hemisphere (Møller 1994; Turner 2006). We measured flight performance traits of barn swallows in a standardized manner in flight tunnels (Rowe et al. 2001; Bowlin and Winkler 2004; Bro-Jørgensen et al. 2007; Matyjasiak et al. 2009; Matyjasiak 2013). We expected a decrease in manoeuvrability, acceleration and velocity with increasing number of feather holes.

**Figure 2.**
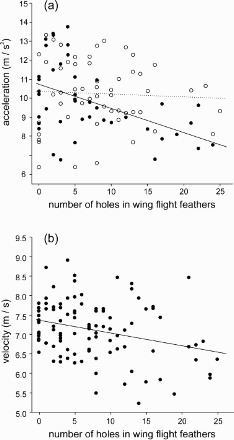
The relationship between maximum acceleration (a) and maximum velocity (b) and the number of feather holes measured on wing flight feathers of the barn swallow. In panel (a), the dotted line and open circles denote males, while the solid line and filled circles denote females. In panel (b), there is no distinction between sexes since the two sexes did not differ. The lines are linear regression lines.

## Methods

### Study site and general methods

The study was carried out in two barn swallow colonies (ca. 20–30 breeding pairs each) located in two nearby horse stables in the Łomianki commune near Warsaw (52° 22′ N, 20° 53′ E, elevation 75 m), central Poland, during the breeding seasons 2010 and 2014. Further details on the study area and the study population are given in Matyjasiak (2013) and Matyjasiak et al. (2013, 2016).

Birds were captured by mist-netting conducted from early spring as they returned from Africa, sexed according to Svensson (1984), ringed with a numbered aluminium leg ring and individually marked with a combination of colour leg rings. Since breeding pairs and unmated males were intensively ringed since 2006, marked birds could be classified as after second year (ASY) – birds in at least their second breeding season, while unmarked birds or birds ringed as nestlings in the previous year were classified as second year (SY) – birds hatched the previous year and in their first breeding season. Since breeding philopatry of this species is very high (Møller 1994; our capture–recapture unpubl. data) this approach is justified.

For each bird a number of biometric measurements were made. The length of wing (from the carpal joint to the tip of the longest primary feather) and outermost tail feathers was measured to the nearest 1 mm with a ruler. Keel and tarsus lengths were measured to the nearest 0.1 mm using a pair of callipers. Body mass was measured to the nearest 0.5 g with a Pesola spring balance. Body mass measurement was repeated on the day of flight tests. Paired measurements were averaged to calculate mean wing, tail and tarsus length. Aspect ratio – an index of the overall shape of the wing – was calculated as (wing-span)^2^/wing area. To measure wing area – an index of the wing size – wing drawings were made by making a tracing of the outlines of fully extended wings. Wing drawings were subsequently analysed in MultiScan ver. 14.02 (Computer Scanning Systems II, Warsaw, Poland). Wing area was estimated as the area of both wings including the area of that part of the body that was included between the wings (Pennycuick 1989). After measurements and ringing the birds were immediately released. The feather hole count was made later, on the day of flight tests (see below). The number of feather holes was counted on flight feathers of the left and right wing and on tail feathers. For this study the feather hole counts for wings and tail were used separately. All measurements were taken by PM.

### Flight performance measurements

Aerial manoeuvrability was tested by releasing birds through a flight maze measuring 18 m × 4 m × 1.6 m (length × width × height). The maze consisted of a metal frame covered in a double layer of fine-mesh garden sunshade netting. The west end of the maze was closed and contained the release box (where the bird was kept before being released in the maze) while the east end was open. The first 9 m section of the maze (on the release box side) was free of obstacles and acted as an acceleration zone. The remaining 9 m section towards the exit acted as a test zone. It contained 16 successive panels of vertical white cotton strings suspended from the roof of the maze and weighed. Both the distance between the strings within a panel and the distances between consecutive panels decreased towards the exit. Further details of the arrangement of the fight maze's test zone used in this study are given in Matyjasiak (2013).

Birds were released (after 2 min of acclimation) from the release box at the closed end of the flight maze and flew through the maze to escape from the open end. The front side of the box was opened remotely with a string. We measured the time taken for a bird to negotiate the test zone of the maze, which was used as measures of the bird's ability to cope with the obstacle course. A faster flight time indicate greater manoeuvrability (Rowe et al. 2001; Bro-Jørgensen et al. 2007; Matyjasiak 2013). Time taken to negotiate the maze test zone was measured based on video images (HDV camcorder SONY HDR-HC1; filming at 25 frames s^−1^) obtained with the use of angled mirrors positioned in line with the first and last panels of strings. A bird's image was reflected in the first mirror as it entered the test section and the second image was reflected in the other mirror when it left the maze. The flight time was determined by counting the number of frames between the two images and converting the result into seconds (accuracy of 0.04 s). Videos were analysed by viewing them frame-by-frame in Edius Pro 3 (Canopus, Reading, UK).

Maximum acceleration and maximum velocity were measured by releasing the birds through another flight tunnel measuring 10 m × 1.2 m × 1.2 m (length × width × height) (Matyjasiak 2013). It consisted of a metal frame covered in a double layer of fine-mesh garden sunshade netting. The covering was opaque. Birds were released from a small release box that was centred on the tunnel's closed end. A Stalker Pro ATS Ka-band radar gun (Applied Concepts Inc., Plano, USA) connected to a Samsung R522 portable computer was mounted on a tripod at the tunnel exit. The radar was run with a minimum speed of 0 and a maximum of 225 kph on High range, with the peak mode off and the auto clear set to 0 s. To minimize signal noise in the radar the flight tunnel was positioned inside an unused building with the open end placed at the exit doors. Radar data were analysed using Stalker Pro ATS ver. 4.5 (Applied Concepts Inc., 2002, Plano, USA), in ‘acceleration run’ mode. The program was configured to discard any data points that occurred before the bird had been released and after it had left the tunnel. To create velocity-versus-time and acceleration-versus-time graphs in ‘acceleration run’ mode we used medium digital filter setting, as recommended by Stalker (Vanman and Shorten 1997). Maximum acceleration and maximum velocity were obtained from these graphs with the graph tracer.

Flight tests were performed in June during the period in which barn swallows feed their first brood nestlings aged 6–15 days. We chose for flight tests clear days with no wind or rain (temperatures of approximately 20–25°C). Birds were captured at dawn, between 0430 and 0530 h. Before flight tests the feather hole count was performed and body weight was measured. We selected for flight tests birds that did not suffer from flight feather breakage. Flight tests were conducted the same morning at 0700–1000 h. First, birds were tested for aerial manoeuvrability in the flight maze and recaptured in a mist-net positioned at the maze exit (the distance between the last panel of strings and the mist-net was ca. 50 cm). Immediately after the manoeuvrability test birds were released through the second tunnel for acceleration and velocity performance, after which they instantly regained freedom. These measurements of flight performance are significantly repeatable, and hence they are sufficiently precise to allow use in statistical analyses (Matyjasiak 2013).

### Statistical analysis

The study sample contained 143 adult barn swallows – 72 in 2010 and 71 in 2014, among which 38 and 36, respectively, were males. No birds in the sample were examined in both years. Since not all birds completed both flight tunnel trials or because the radar recordings not always were reliable, sample sizes for the analyses of specific flight performance variables varied compared to the above value. Six birds failed to complete the flight maze trial – these birds hovered and/or circled in the test zone or perched on strings rather than flying through the maze. We were unable to obtain flight velocity data for 44 swallows and flight acceleration data for 46 swallows – these birds hovered and circled within the tunnel before they flew out (44 cases) or they were indistinguishable from background noise on radar during the initial (about 0.5 s) phase of flight (2 cases).

The feather hole counts were not significantly correlated with morphological predictor variables (r coefficients lower than 0.15, all *p*s > 0.05), including the tail length, which is an indicator of quality of male barn swallows (the correlation between male tail length and the number of holes in wing feathers: *r* = −0.10, *t* = −0.87, n = 73, *p* = 0.39; or the number of holes in tail feathers: *r* = −0.15, *t* = −1.34, n = 73, *p* = 0.18). Furthermore, these variables were not associated with body condition index of individual barn swallows (general linear model with either the number of holes in wing feathers or the number of holes in tail feathers as dependent variables, and body mass at the day of flight tests and keel length as independent variables; the number of holes in wing feathers: $R_{\mathrm{adj}.}^{2}$ = 0.003, d.f. = 2, 140, *F* = 0.22, *p* > 0.1; the number of holes in tail feathers: $R_{\mathrm{adj}.}^{2}$ = 0.01, d.f. = 2, 140, *F* = 0.80, *p* > 0.1). The sexes did not differ with respect to the numbers of wing or tail feather holes (wing feather holes: *F* = 0.68, d.f. = 1, 141, *p* > 0.1; tail feather holes: *F* = 0.33, d.f. = 1, 141, *p* > 0.1). Morphological, flight performance, and feather hole count variables were log transformed before analysis.

We tested whether the number of flight feather holes predicted the flight performance traits using a general linear model. We ran models of each of the three flight performance traits (aerial manoeuvrability, maximum acceleration and maximum velocity) as a function of the number of holes in wing feathers and the number of holes in tail feathers as predictor variables. The initial model contained also covariables – age, sex, year, colony, wing area, aspect ratio, tail length, keel length and body weight on the day of the flight tests. Insignificant variables were removed from the models, which were then rerun. However, those variables that significantly interacted with other variables in the model were not removed regardless of their significance. We checked the residuals for normality and homoscedasticity at each step of model reduction. Statistical analyses were made in STATISTICA ver. 12 (Statsoft Inc., Tulsa, USA).

## Results

In our final general linear model, time to copy the flight maze was not significantly related to the number of feather holes in the wing flight feathers or in the tail feathers (Table 1). This suggests no significant effect of the number of feather holes on aerial manoeuvrability. In this model manoeuvrability increased significantly with increasing tail length and decreasing aspect ratio (a proxy for wing shape; Table 1). In general linear model with maximum acceleration as dependent variable, this flight trait was significantly negatively associated with the number of holes present in the wing flight feathers, and this relationship was more pronounced in females than in males (significant interaction wing feather holes × sex; Table 1, Figure 2(a)). The final model accounted for 17% of the variation in acceleration performance. The number of feather holes explained 4.1% of the variation. The sexes did not differ significantly in maximum acceleration. Maximum acceleration was not significantly related to the number of holes in tail feathers. This flight variable was significantly negatively associated with aspect ratio and positively with keel length, with the latter being a proxy for body size (Table 1). Because of the significant interaction we ran separate analyses for males and females. Female acceleration performance was significantly negatively associated with the number of holes in wing feathers (*r*^2^ = 0.22, d.f. = 1, 40, *F* = 11.07, *p* = 0.002, slope = −0.35, 95% *CI* −0.62 to −0.07) and was significantly positively associated with keel length (*r*^2^ = 0.12, d.f. = 1, 40, *F* = 5.26, *p* = 0.027, slope = 0.31, 95% *CI* 0.03 to 0.59). The model accounted for 28% of the variation in acceleration performance of females ($R_{\mathrm{adj}.}^{2}$ = 0.28, d.f. = 2, 40, *F* = 9.16, *p* < 0.001), and the number of feather holes explained 18.7% of the variation. In contrast, male acceleration performance only was significantly negatively related to aspect ratio (slope = −0.28, 95% *CI* −0.54 to −0.01, *t* = −2.08, *p* = 0.04; overall model $R_{\mathrm{adj}.}^{2}$ = 0.06, d.f. = 1, 52, *F* = 4.31, *p* < 0.043).

**Table 1.** General linear models of flight performance traits of barn swallows (aerial manoeuvrability, maximum acceleration and maximum velocity) as a function of numbers of feather holes, with sex and morphological traits as covariables. Time to copy the flight maze was used as a measure of aerial manoeuvrability, with a shorter flight time indicating grater manoeuvrability. The number of feather holes in tail feathers was not significantly related to flight performance traits and consequently is not shown in the table.

Effects	Time to copy the flight maze		Acceleration		Velocity	
	SS	Test statistics (slope, 95% *CI*)	SS	Test statistics (slope, 95% *CI*)	SS	Test statistics (slope, 95% *CI*)
Constant	0.028		8.216	* *	0.012	* *
Wing feather holes			13.501	*F*_1,91_ = 4.82* (−0.208, −0.397 to −0.019)	4.412	*F*_1,95_ = 9.18** (−0.283, −0.468 to −0.097)
Wing feather holes × sex			14.563	*F*_1,91_ = 5.19* (0.328, 0.042 to 0.614)		
Sex			1.395	*F*_1,91_ = 0.50 (−0.108, −0.411 to 0.196)		
Wing area					2.233	*F*_1,95_ = 4.65* (0.203, 0.016 to 0.389)
Aspect ratio	0.793	*F*_1,132_ = 5.95* (0.207, 0.039 to 0.375)	21.514	*F*_1,91_ = 7.67** (−0.276, −0.473 to −0.078)		
Keel			11.389	*F*_1,91_ = 4.06* (0.204, 0.003 to 0.406)	2.212	*F*_1,95_ = 4.60* (0.202, 0.015 to 0.389)
Tail length	0.802	*F*_1,132_ = 6.01* (−0.208, −0.376 to −0.040)				
Model statistics	$R_{\mathrm{adj}.}^{2}$ = 0.06, *F*_2, 132_ = 5.14**		$R_{\mathrm{adj}.}^{2}$ = 0.17, *F*_5,91_ = 4.81***		$R_{\mathrm{adj}.}^{2}$ = 0.15, *F*_3, 95_ = 6.75***	

SS – sums of squares; *p < 0.05, **p < 0.01, ***p < 0.001, blank – not significant.

Maximum velocity was significantly negatively associated with the number of holes in wing feathers, and this relationship was independent of sex (Table 1, Figure 2(b)). The final model accounted for 15% of the variation in maximum velocity, with the number of feather holes explaining 8.1% of the variation. Moreover, this flight trait was significantly positively associated with wing area (a proxy for wing size) and keel length (Table 1). Maximum velocity was not significantly related to the number of holes in tail feathers.

## Discussion

In the present study of barn swallows we found that two short-term flight performance traits – maximum acceleration and maximum velocity – were significantly negatively associated with the number of holes present in the wing flight feathers. The number of holes present in the tail feathers was not significantly associated with any of these flight variables. In the case of acceleration, the negative relationship between the number of feather holes and flight performance was sex specific. While acceleration significantly decreased with increasing wing feather hole numbers in females, there was no such significant association in males. To our knowledge, this is the first study to show the existence of a relationship between flight performance and the number of feather holes. Furthermore, we found that aerial manoeuvrability was not significantly associated with the number of holes, whether in the wing flight feathers or in the tail feathers.

Flight performance trials used in our study assumed that birds were escaping from flight tunnels. In the case of flight maze, the crowded stringed section forced the birds to perform increasingly tight turns around obstacles. Flight performance displayed by the birds in such conditions may be close to the maximum of manoeuvrability and acceleration (Lind 2001; Rowe et al. 2001; Bowlin and Winkler 2004; Bro-Jørgensen et al. 2007). Short-term flight performance traits analysed in this study, especially aerial manoeuvrability and maximum acceleration, are expected to be important for foraging efficiency of aerial insectivores (Waugh 1978), and also for predator avoidance and long-distance flight efficiency in birds in general (Metcalfe and Ure 1995). The results of our study may suggest that holes occurring in wing feathers can impair flight acceleration and velocity, especially in the case of females. This effect seems weak (4.1% of variation in maximum velocity can be attributed to the impact of feather holes) to moderate (18.7% of variation in the case of maximum acceleration). The higher detrimental effect of holey feathers on maximum acceleration in females can be explained by morphological differences between the sexes: compared to males, female barn swallows have shorter, lower aspect ratio wings and higher wing loadings (Møller et al. 1995; our unpubl. data). These morphological characteristics of wings may increase the susceptibility of females to feather deformities that impair aerodynamic performance of wings. Female barn swallows seem generally more sensitive than males to factors that increase aerodynamic costs of flight (e.g. Pap et al. 2005; Scandolara et al. 2014).

Our study is non-experimental, however, and alternative explanations of the results cannot be excluded. According to an alternative scenario, the correlation between the number of feather holes in wing feathers and acceleration and velocity may result from the fact that poor quality individuals with low quality plumage are worse fliers and, due to compromised plumage, are more susceptible to structural damage of flight feathers. Consequently, there may be no causal relationship between the number of feather holes and flight performance. This scenario does not seem to be supported by the results obtained in this study. First, we found no significant relationship between the number of feather holes and condition indices of male and female or tail length of male barn swallows. Second, we found no significant relationship between the number of feather holes and wing size or aspect ratio. The latter result may suggest that there is no relationship between the quality of flight feathers and the number of holes in these feathers. We would like to suggest, however, that future studies should take into account measures of feather microstructure (e.g. Pap et al. 2015) in analyses of the effect of holey feathers on flight performance.

Results obtained in this study are consistent with the results of other studies on barn swallows, which showed that feather holes had a significant effect on migration efficiency and fitness, as males and females with more feather holes delayed arrival from spring migration and survival rate of females was significantly reduced (Møller et al. 2004a; Pap et al. 2005). However, it should be noted that a recent study on another swallow species, the tree swallow *Tachycineta bicolor*, reported no effect of feather holes on migration performance or annual survival (Lombardo et al. 2015).

The mechanism behind the negative association between the feather hole intensity and flight performance is unclear. One possible explanation is that holes may worsen air tightness of flight feathers, which in turn may affect the aerodynamic performance of these feathers (Videler 2005). A future study might test for the effect of feather holiness on the transmissivity and aerodynamic performance of wing flight feathers and wings (Müller and Patone 1998; Heers et al. 2011). Surprising is the fact that the number of feather holes showed significantly negative associations with flight performance in tests of acceleration and velocity and no such associations in tests of aerial manoeuvrability. A possible explanation for this discrepancy is that during manoeuvrability trials the wings of tested birds did not work at maximum of their aerodynamic capacity. While acceleration and velocity measurements were performed when birds accelerate, manoeuvrability measurements took place when the birds left the acceleration zone of the maze.

In conclusion, two short-term flight performance traits in barn swallows – maximum acceleration and maximum velocity – were significantly negatively associated with the number of feather holes occurring in wing flight feathers. We found stronger negative effect of feather holes of acceleration in females than in males. These relationships are consistent with the hypothesis that feather holes are costly in terms of impaired flight. A future study on this issue could take into account the independent effect of feather microstructure on flight performance. Also, the exact mechanism behind the negative association between the number of feather holes and flight traits remains to be investigated.
